# OSeMOSYS Global, an open-source, open data global electricity system model generator

**DOI:** 10.1038/s41597-022-01737-0

**Published:** 2022-10-14

**Authors:** Trevor Barnes, Abhishek Shivakumar, Maarten Brinkerink, Taco Niet

**Affiliations:** 1 Sustainable Energy Engineering, Simon Fraser University, 10285 University Dr, Surrey, BC V3T 0N1 Canada; 2 Centre for Environmental Policy, Imperial College London, 16-18 Prince’s Gardens, London, SW7 1NE United Kingdom; 3 STEER Centre, Department of Geography and Environment, Loughborough University, Epinal Way, Loughborough, LE11 3TU United Kingdom; 4 MaREI, the SFI Research Centre for Energy, Climate and Marine, Environmental Research Institute, University College Cork, 6 Lee Rd, Cork, T23 XE10 Ireland; 5 School of Engineering, University College Cork, College Road, Cork, P43 C573 Ireland; 6 Thayer School of Engineering, Dartmouth College, 15 Thayer Dr, Hanover, NH 03755 USA

**Keywords:** Energy modelling, Energy policy, Energy economics

## Abstract

This paper describes OSeMOSYS Global, an open-source, open-data model generator for creating global electricity system models for an active global modelling community. This version of the model generator is freely available and can be used to create interconnected electricity system models for both the entire globe and for any geographically diverse subset of the globe. Compared to other existing global models, OSeMOSYS Global allows for full user flexibility in determining the time slice structure and geographic scope of the model and datasets, and is built using the widely used fully open-source OSeMOSYS energy system model. This paper describes the data sources, structure and use of OSeMOSYS Global, and provides illustrative workflow results.

## Introduction

There is growing urgency in identifying development paths to decarbonize our energy system while maintaining global economic and social well-being. This has brought about a debate amongst scientists and economists on the best approaches for reaching these goals, but there is broad consensus that variable renewable energy resources, such as solar and wind generation, will play a major role in the transition. To address the challenges that variability imposes on the global energy system a variety of potential solutions have been suggested, from deploying large scale electricity storage^1^ to inter-connecting power systems globally to allow excess in one area to be sent to areas with deficit^2^. In either of these cases (or others such as power-to-x, fuel switching, deep demand-side management, etc.), there is a trade-off between the costs and impacts of the infrastructure required and the benefits the solution brings. Active open-source (and closed) Energy system models have the proven ability to provide insights in these dynamics and to inform energy and climate policy globally^3^. It is our aim to develop accessible code and workflows that can be continuously improved and injected into an active global user base given the urgency of the climate challenge.

This paper describes OSeMOSYS Global, an open-source, open-data, freely available global model generator that can be used to quickly and efficiently create interconnected or standalone, geographically diverse, electricity system models to address the trade-offs and synergies inherent in deploying renewable energy and expanded transmission capacity at scale across large geographic areas. OSeMOSYS Global is designed to generate fully functional electricity sector models. Through building a flexible workflow on top of the **O**pen **S**ource **e**nergy **MO**delling **SYS**tem (OSeMOSYS) framework^4–6^ (https://github.com/OSeMOSYS), this acts as the first step in creating a full global energy system model generator which will incorporate numerous energy sectors. OSeMOSYS Global’s workflow is developed with user accessibility in mind. To run a scenario, the user only needs to interface with a single configuration file, keeping the barrier of entry to begin using OSeMOSYS Global low. While being comfortable with Python, command line interfaces, and OSeMOSYS can be beneficial, OSeMOSYS Global is easily accessible for users with minimal to no prior knowledge in these areas.

## Background

In recent years a range of global energy system models have been introduced^1,7–12^, however, there are a range of limitations in these studies and models. First of all, many models have strong limitations in spatial detail^9–11^. This is particularly problematic in understanding the role of international electricity trade when designing decarbonization policies, as the spatial sensitivity tests should be done to properly evaluate trade results^13^. Secondly, the majority of tools are not openly available in the public space^1,7,11^ or the software used is not available freely except for academic use^8^. Creating an open source energy model comes with numerous benefits, including increasing the transparency of results and being able to leverage a community of experts to contribute towards the model^14,15^. These benefits help ensure modeling errors and biases are reduced and assumptions can be clearly documented, as anyone is free to investigate the code and submit fixes to the workflow. OSeMOSYS in particular has been the subject of scores of reviewed academic analyses. In one systematic literature review, 17% of national energy system modelling decarbonisation analyses were undertaken with OSeMOSYS^3^, and it has been identified in a peer reviewed analysis as one of only four open source tools that are mature enough for policy analysis^16^. Finally, few tools allow for quick model generation to perform scenario exploration and evaluation over changing model assumptions^12,17^. Scenario exploration is often required to ensure a solution is stable and realistic over the given variety of modeling assumptions, such as spatial and temporal modeling resolution, interconnection options, technology choices, and emission penalties. Implementing a system to quickly explore scenarios and perform sensitivity checks in an organized manner will be invaluable to modellers and researchers. OSeMOSYS Global addresses these limitations by being a truly open-data and open-model generator, while also allowing for flexible geographic scopes and easy-to-define user configurable modeling assumptions and parameters.

The current literature shows two existing open-source global model generators with different accessibility, scope, and state of development. Mattsson *et al*.^12^ introduced SuperGrid.jl (https://github.com/niclasmattsson/Supergrid), a novel automated model generator restricted to capacity expansion of the global electricity (rather than energy) system. It does so also supporting the automated generation of electricity system input data for any arbitrary world region (https://github.com/niclasmattsson/GlobalEnergyGIS). However, SuperGrid.jl has not been the subject to accessible peer review, nor is the formulation described in plain English nor mathematics, and it has no documented community. As an electricity-system only model, potential extensions to include power-to-X (including hydrogen integration) or fuel switching are limited. The other noteworthy project is the PyPSA Meets Africa model^17^ which is an open optimization model of the African transmission system built on top of the Python for Power System Analysis package^18^ (PyPSA, https://github.com/PyPSA). While the African model is already released, expansion towards a fully functioning global electricity PyPSA model, coined PyPSA-Earth, is currently under development. Moreover, plans to release a global sector coupled model are expected for 2023 and onwards.

Also of note are recently released “starter kits” published by Allington *et al*.^19^. These kits include energy system data for a number of developing countries which can be imported easily into the OSeMOSYS framework. While beneficial for providing policy makers in specific countries a starting point to build their own country-level energy system models, the starter kits do not have full global coverage and are not classified as model generators allowing for quick scenario evaluation.

The work introduced in this paper focuses on the development of a model generator with the intent to extend coverage to the full global energy system - for easy access to a global community. This is done via the adoption of OSeMOSYS, a proven and widely used open-source modeling framework^4,5^ in terms of research application and capacity building efforts, and the work is positioned to be well supported by a large community of users.

## Results

**Fig. 1:**
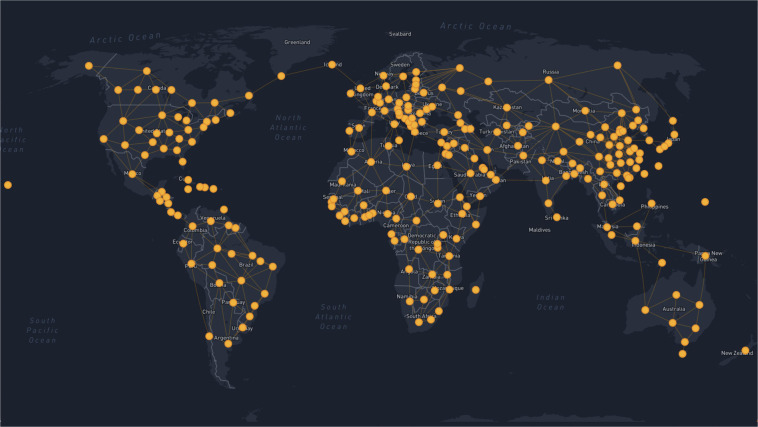
Nodes available for model generation in OSeMOSYS Global. Each node represents the geographic centroid of a modeled region. For further information about regional boundaries, see^8^.

OSeMOSYS Global is designed to be used as a model generator to support long-term energy system decarbonization efforts. This model generator allows the user, through the automated running of Python scripts and shell commands, to assemble the necessary input data and create an electricity system model that covers a user-specified geographic scope. This scope can range from areas as small as a country/region to as large as the entire globe, with all 265 available nodes shown in Fig. 1. The automated workflow creates an OSeMOSYS compatible electricity system model that can be used for transmission and renewable energy deployment scenario evaluation in context of the wider energy system.

In this section we highlight the flexibility of OSeMOSYS Global through showcasing a variety of scenarios created for different geographic scopes and temporal scales. All result figures displayed in this section are automatically generated by the workflow and can be easily recreated by readers through changing a configuration file. The results displayed and discussed in this section showcase the flexibility and applicability of the model generator workflow as a proof of concept; further work is required to perform a full range of spatial temporal sensitivity tests and use OSeMOSYS Global to answer specific policy questions, both of which are beyond the scope of this methodology focused paper.

### Flexible spatial resolution

OSeMOSYS Global can be used as a model generator to carry out power system capacity expansion analysis of different geographical regions in the world. To showcase this functionality, we run the model for three different geographic scopes; a system with only India (five sub-country nodes), a system including Bangladesh, Bhutan, Nepal, and India (BBIN), and a world system that includes all available 164 countries separated by 265 nodes

In each scenario the model horizon is set to 2015–2050 with each year divided into 8 representative time periods, four seasons each with a 12 hour day/night cycle (see Methods for more detail on the time slice structure). Moreover, other configurable parameters applied include a carbon tax of $50 per Tonne of CO2 and regional trade of commodities and electricity. The model runs in this example do not allow new investment in geothermal, biomass, concentrated solar power, and wave energy sources, though existing plant capacities for these technologies are included as residual capacity. Results from OSeMOSYS Global do not yet include energy storage functionality and are later discussed in future work opportunities. To generate each scenario, the global model is partitioned according to the user-defined configuration file. Finally, the seventeen power generation technologies available to the model are summarized in Table 1.

**Table 1:** Power Generation Technologies.

Technology	Code
Biomass	BIO
Combined Cycle Natural Gas	CCG
Coal	COA
Cogeneration	COG
Concentrated Solar Power	CSP
Geothermal	GEO
Hydroelectric	HYD
Open Cycle Natural Gas	OCG
Oil	OIL
Other	OTH
Petroleum	PET
Solar Photovoltaic	SPV
Nuclear	URN
Wave	WAV
Waste	WAS
Offshore Wind	WOF
Onshore Wind	WON

The number of automatically generated nodes, technologies, and commodities in each scenario run are displayed in Table 2. Approximate solve times for each scenario are also shown, which were obtained using machines equipped with Intel Core i9-9900 CPU’s, 64 GB of RAM, and SSDs.

**Table 2:** OSeMOSYS Global Geographic Scenario Data.

Scenario	Nodes	Technologies	Commodities	Solve Time
India	5	165	76	1 min via CBC
BBIN	8	279	131	3 min via CBC
World	265	9438	4127	5 hrs via Gurobi

A note should be made on the capability of open-source solvers to solve models generated from OSeMOSYS Global. Due to its flexible workflow, it is possible to create models that exceed the current limits of open-source solvers on reasonable hardware. Solving the World scenario using the CBC solver did not produce a result in a reasonable amount of time (less than 100 hrs). This makes using CBC infeasible for policy analysis scenarios at the global scale, though we expect it would have found a solution if left to run for long enough. In these cases, commercial solvers such as Gurobi or CPLEX can be selected through the configuration file. While this does restrict the openness of the workflow, these solvers are free for academic use. Moreover, the capability of open-source solvers is not a problem unique to OSeMOSYS Global^20^, and there is ongoing development of open source solvers that may ameliorate this issue, such as the HiGHS solver^21^.

**Fig. 2:**
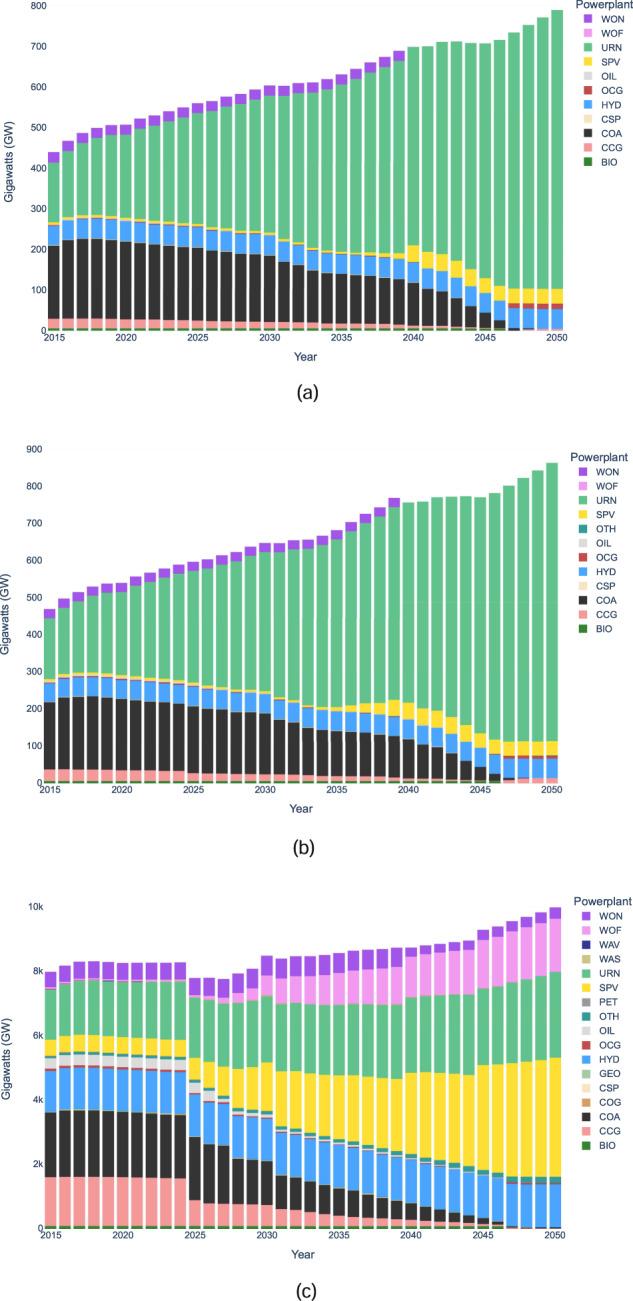
System Capacity Results for (**a**) India (**b**) BBIN (**c**) World.

**Fig. 3:**
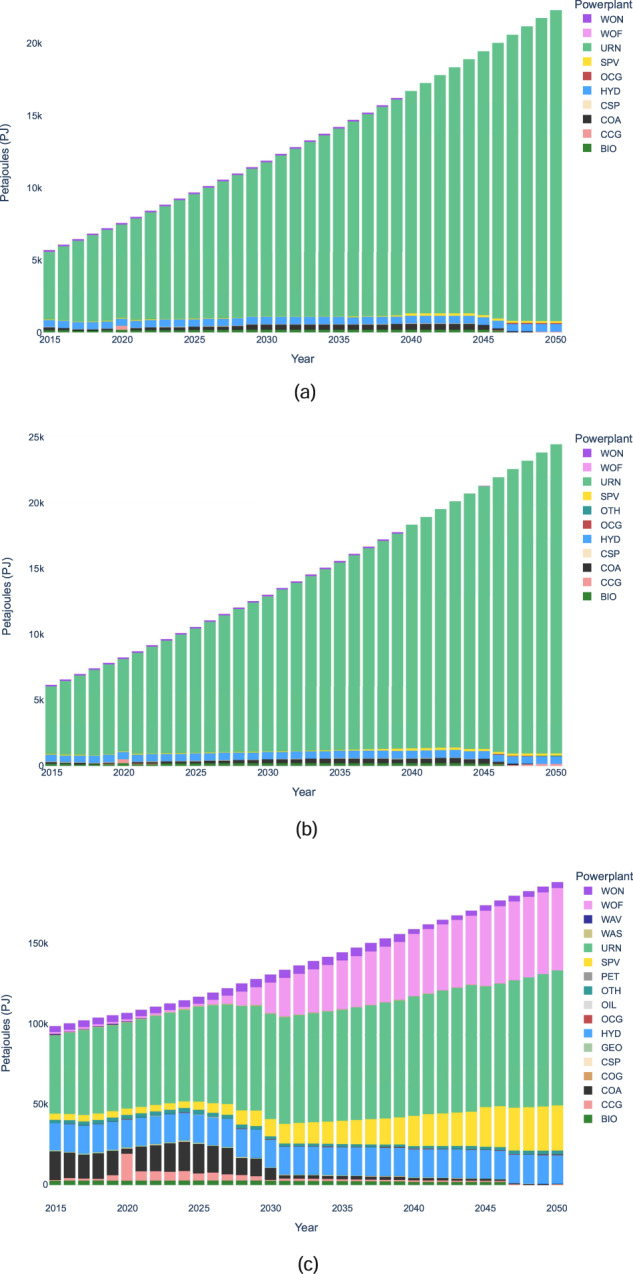
System Generation Results for (**a**) India (**b**) BBIN (**c**) World.

System capacity and generation results for all three scenarios are shown in Figs. 2 and 3 respectively. Notably, the energy mix differs drastically when comparing the world scenario to the India and BBIN scenarios. For example, in 2050 the world system’s generation mix consists of a variety of generation sources, primarily wind, solar, nuclear and hydro, while the India and BBIN results are dominated by nuclear. While the energy supply mix results may need further analysis, such as removing technology bias though implementing resource limits on nuclear in BBIN, they are only intended to show the methodology of OSeMOSYS Global. The results presented in this paper should be interpreted as results caused by changing model parameters to test any modeling assumptions made. Moreover, results from OSeMOSYS Global can help identify potential avenues for continued policy research, such as how regional trade and carbon tax factors contribute to variable renewable energy investment pathways, and modelling research questions, such as evaluating the geographic sensitivity of the results.

Repetitively creating scenarios of different geographic scopes in an accurate and efficient manner is a key strength of OSeMOSYS Global. Geographic sensitivity studies help validate model results by testing how stable the solution is with different spatial extents and spatial aggregation assumptions^13,22^. However, collecting and formatting data to perform these sensitivity tests are often tedious and can easily result in data transcription errors. OSeMOSYS Global will perform this data collection and processing work simply by updating a configuration file. This allows the modeller to focus on understanding the system response based on changing model assumptions, rather than finding difficult data related bugs.

### Interconnection analysis

**Fig. 4:**
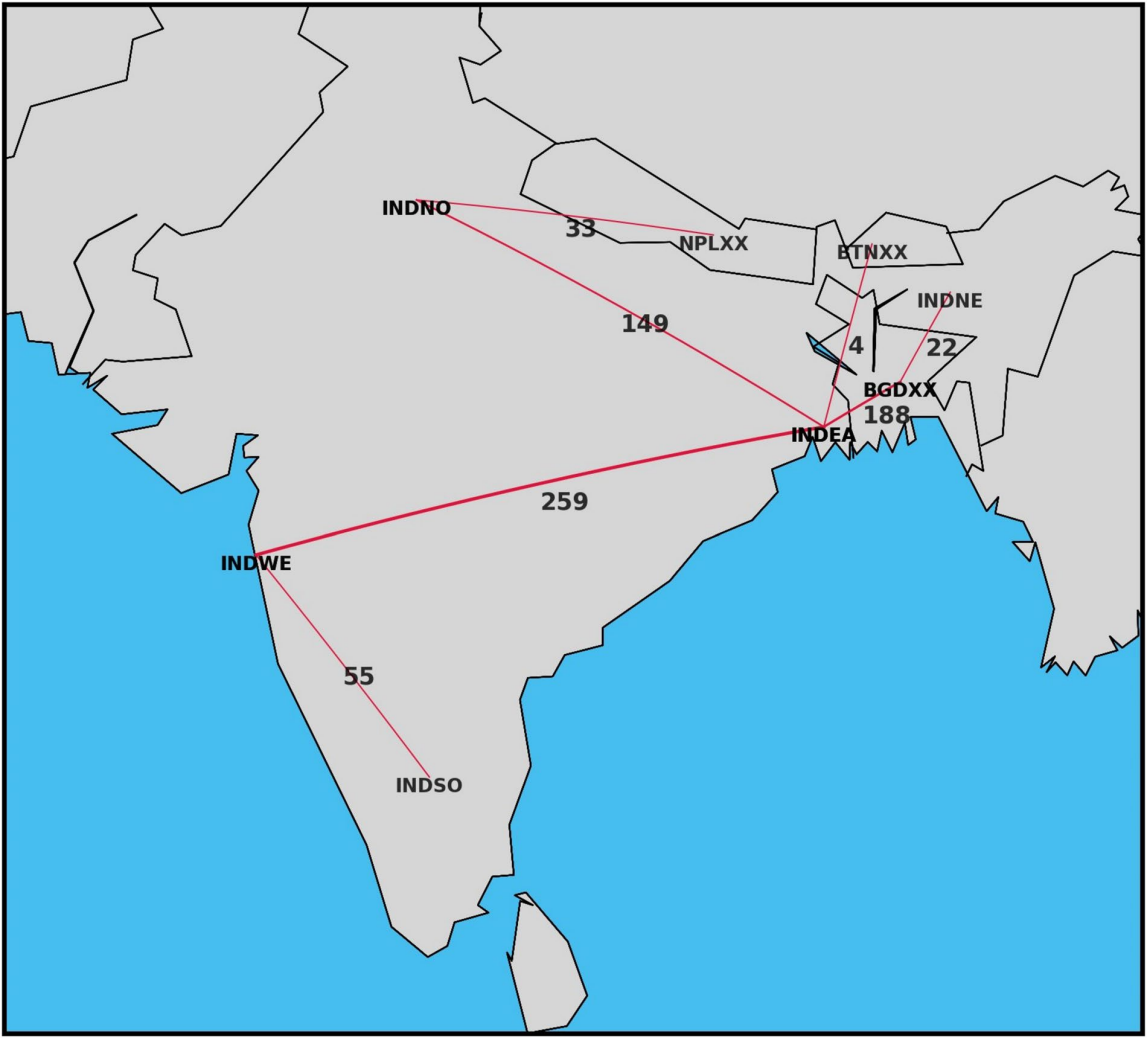
BBIN Total Electricity Trade Results for 2050 (PJ).

Reaching decarbonisation targets is expected to require significant investment in variable renewable generation sources, which provides an opportunity to increase transmission capacity to help balance loads and demand across larger regions^2^. For example, geographic areas that have excellent solar irradiation and wind speeds are often not close to where peak demand centers are. Increasing transmission capacity between these locations can be a cost effective way to increase renewable generation in the system. OSeMOSYS Global allows users to easily visualize the amount of electricity trade occurring between multiple nodes, as shown in Fig. 4.

Investigating the flow of energy trade helps users understand system bottlenecks and opportunities. For example, comparing the system generation results for India to BBIN (Fig. 3a,b) shows minimal change, likely due to the significantly larger size of India. That said, to evaluate the potential of system cooperation through analyzing regional trade, we can see how efficient renewable resources (predominantly hydro) externally can assist with India’s growing demand and vice versa.

### Flexible temporal resolution

Selecting the correct temporal representation to effectively answer a research question is difficult, with numerous papers discussing the importance and challenges of this task^13,23,24^. Especially in systems with large shares of variable renewable energy sources, capturing the intermittent nature of the sources is often needed to obtain credible results. However, much like with the geographic resolution, performing sensitivity analyses to test a model with changing temporal resolutions in a repeatable and accurate way is difficult. OSeMOSYS Global allows the user to change between 1 and 288 time periods per year, over a maximum model horizon of 2015–2100 (see Methods for more details on time slicing options).

**Fig. 5:**
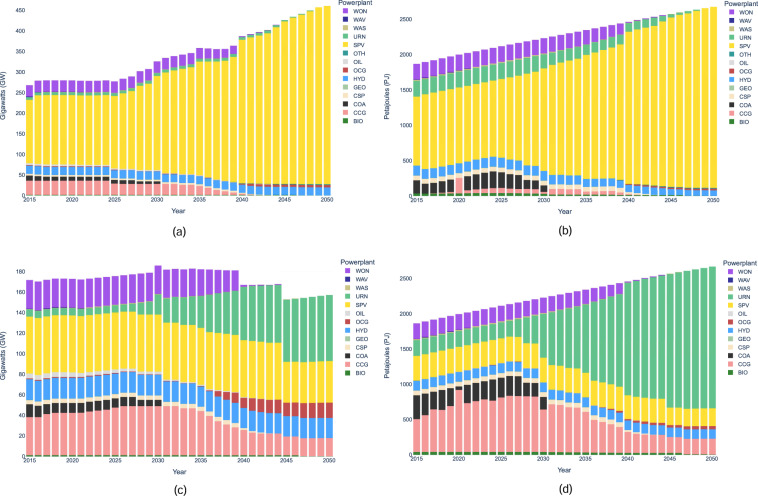
Spain and Portugal Power System Results. Default Temporal Resolution (**a**) System Capacity (**b**) System Generation. Increased Temporal Resolution (**c**) System Capacity (**d**) System Generation.

To clearly demonstrate the importance of repeating scenario runs with changing temporal resolutions, a sample case of Spain and Portugal is run. Figure 5 showcases the capacity and generation results of this study at two different resolutions over a model horizon of 2015–2050. The first scenario has 4 seasons, each with a 12 hour day/night cycle, producing 8 time periods per year. The second scenario has 12 seasons, each with twelve 2 hour time segments, representing 144 time periods per year. The resulting changes are drastic, with the reliance on solar power significantly reducing in the higher temporal resolution graphs. Even with the region’s excellent solar potential, due to storage not being introduced into the scenarios the system has no flexibility options apart from regional trade. This means if there is excess solar generation during the day, there is no way to store it and release it overnight during periods of minimal generation. Therefore, it favours dispatchable generation sources, such as nuclear and natural gas, which can consistently be used to meet base load.

**Fig. 6:**
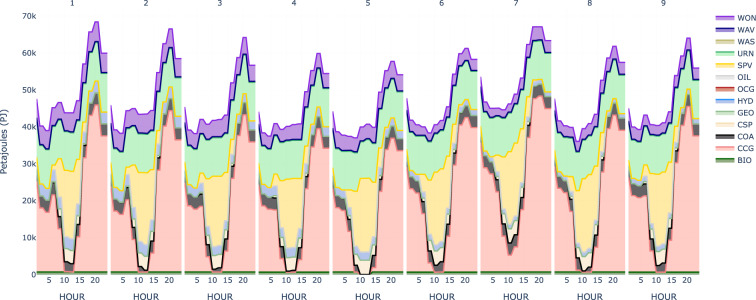
Spain and Portugal Power System Hourly Generation Results in 2030.

Understanding what generation technologies contribute to meeting the demand for each time segment is often a key result to report on when completing energy system studies. Therefore, OSeMOSYS Global provides functionality to view hourly results to analyze and evaluate the generation mix and finer scales then just over the year. Figure 6 displays the hourly higher temporal resolution scenario results for Spain and Portugal. From this figure, it is clear that while wind and solar are consistently contributing to the demand, there is also a constant supply from nuclear to help meet base load, highlighting the need for consistent dispatchable supply. Moreover, the daily spikes in load, around 5 pm, showcase a period when load increases but solar generation is starting to diminish. This leads to a short, but significant, production ramp up from natural gas generators to alleviate the energy mismatch. The ability of OSeMOSYS Global to quickly change temporal resolution allows for more nuanced analysis than using a model with fixed temporal resolution. One could, for example, run the model with a coarse temporal resolution to get some rough estimates for calibration/test operation and then run the model with much finer temporal resolution to evaluate the operational details.

## Discussion

This paper showcases the flexible workflow of OSeMOSYS Global, an open-source, open-data, freely available global electricity system model generator. However, it is also helpful to highlight some specific use cases for which OSeMOYS Global can be applied and to discuss future development pathways. This section will do both.

### Potential applications

OSeMOSYS Global allows for flexible and fast scenario exploration studies. Its ability to perform model optimizations with different spatial and temporal resolutions positions it well to investigate a wide range of energy policy research questions.

As a global model with high spatial resolution, concepts such as the global grid^25^ can be more quantitatively assessed. Current studies of the global grid lack high spatial resolution due to limitations in the available modelling^2^. OSeMOSYS Global has the ability to fill this significant research gap by providing an open-source set of tools to perform global system integration studies at both high temporal and spatial resolution.

If a research question involves performing studies in key regional areas, such as load shifting in South-East Asia, solar studies in GCC India, hydroelectric studies in Sub-Saharan Africa, or wind studies in the North Sea region, OSeMOSYS Global can quickly perform model runs with different modelling assumptions, such as a higher or lower carbon tax or with scaling technology cost parameters. This can provide insights into what policy paths deserve and require further investigation.

At a country level, OSeMOSYS Global can be a valuable tool to assess different technology investment options. For example, questions such as whether it would be more cost efficient for the New England system operator to make use of its untapped offshore wind resources or to extend its transmission integration to Canada or other power systems within the US could be answered. Lastly, it’s worth highlighting that due to the spatial flexibility of OSeMOSYS Global one could envision performing studies in an iterative fashion where runs with higher spatial detail could provide boundary constraints for runs with lower spatial detail but enhanced temporal and technological detail and vice versa.

### Future development

A number of improvements are available to enhance the functionality of OSeMOSYS Global from a power system perspective. Incorporating natural resource potentials for both biomass and geothermal potential, either through the use of geospatial data or a new openly available dataset, may diversify the dispatchable generation sources suggested. Results from weather models can be used to generate capacity factors for wind, solar, and hydroelectric profiles given differing climate scenarios at different model horizons. Short-term storage options, such as electric batteries, can improve the evaluation of variable renewables on the system, while technologies that enable long-term storage, such as pumped hydro storage, can improve the assessment of the impact of seasonal variations. Providing the user options to select different time slicing approaches, such as representative days or clustering methods, can help test temporal assumptions and better represent time-dependent input data^26^. Finally, demand projections can be improved to account for increased electrification over the next decades as decarbonization policies become more aggressive.

Beyond the electricity system, other energy sectors can be built into OSeMOSYS Global to transform it from a power system model to a full energy system model. For example, current research suggests that a closer integration of the gas and electrical grids will help improve decarbonization efforts^27,28^. The OSeMOSYS framework natively supports sector coupling technologies and multiple end use energy carriers, as demonstrated in numerous studies^9,29,30^. Therefore, a full energy system representation can be built on top of the existing OSeMOSYS Global workflow without altering the underlying OSeMOSYS framework. Additionally, emerging technologies, such as hydrogen electrolyzers and fuel cells, can be added to evaluate the role specific technologies will have on decarbonizing the whole energy system. Extending beyond the energy system, environmental indicators and factors can also be implemented into OSeMOSYS Global’s workflow. The Climate, Land, Energy, and Water modelling framework (CLEWs)^31^ (https://github.com/OSeMOSYS/CLEWs) is an extension of OSeMOSYS that allows researchers to investigate the implications of decarbonization scenarios in more than just the energy sector. For example, tracking the water requirements to irrigate crops used in biofuel production can give insights into the feasibility of the fuel source. Developing a CLEWs Global workflow on top of the OSeMOSYS Global workflow will further expand the breadth of research questions the model generator can be used for.

Finally, linking OSeMOSYS Global to a more detailed power system model can give insights into the operational feasibility of the results. Due to the coarse time slicing requirements needed to remain computationally tractable over a long time horizon, much of the detailed power flow analysis is abstracted out in OSeMOSYS. Other open source frameworks, such as PyPSA, address this by including operational constraints at a transmission system level. Creating an automated link between OSeMOSYS Global and operational power system models, such as PLEXOS-World^8^ or with the PyPSA Meets Earth Model^17^, can create a workflow that has the best of both worlds; a long-range planning model with the ability to test operational feasibility. An example application of this is introduced in^32^ where OSeMOSYS Global has been soft-linked to PLEXOS-World to assess the operational performance of a subsea interconnector between Oman and the west of India.

## Methods

OSeMOSYS Global is a workflow management system that executes a series of Python scripts and terminal commands in a specific order to create, solve, and visualize the results from an OSeMOSYS model. This section will describe how the workflow is constructed, how the scripts and commands behind the workflow function, and what data is used in the workflow.

### Workflow

Creating a flexible system for policy makers and researchers to investigate decarbonization pathways at differing time and geographical scales is a key component of OSeMOSYS Global. This requires a workflow that will dynamically update with each scenario run. To generate scenarios, users modify a single configuration and execute the workflow, providing a low barrier of entry that requires minimal to no prior Python and OSeMOSYS knowledge. Moreover, as OSeMOSYS Global grows and develops, having a mechanism to easily add in new user defined assumptions, such as technology retirement requirements or emission limits, will be required. A popular open-source tool used to manage these types of flexible workflows is the Python package Snakemake^33^.

Snakemake provides a system to create scalable and flexible workflow pipelines using Python syntax. In Snakemake, rules are defined which have input files, output files, and an associated script or shell command. The input and output files can either have a specific filename or a generic filename, allowing for reusable rules. Based on the defined input and output files, Snakemake will determine how the rules are linked and execute them in the correct order, parallelizing the work where possible. Furthermore, Snakemake will search through the workflow before running to only execute rules downstream of where changes have been made. This can save significant processing time, as during scenario iteration the workflow will skip initial data processing steps if changes only occur to data at a later stage in the workflow.

**Fig. 7:**
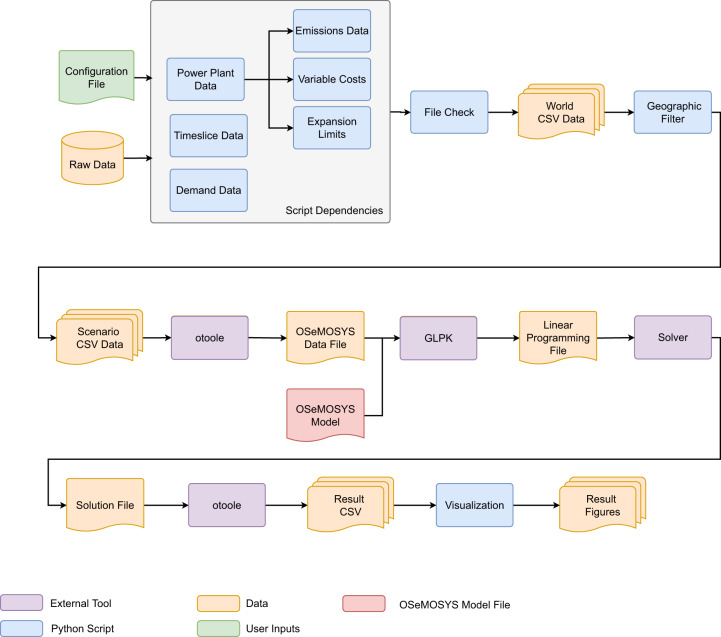
OSeMOSYS Global High Level Workflow.

The high-level overview of the OSeMOSYS Global’s Snakemake workflow is shown in Fig. 7 below, and later discussed in more detail. The user starts by inputting parameters into the configuration file and running the workflow, which will execute all steps shown automatically. To start, Python scripts process the raw data into formatted scenario data. The Python package otoole is then used to create an OSeMOSYS compatible data file, which is combined with the OSeMOSYS model file to create a solver agnostic linear programming file. The model is solved and the results are processed and visualized. While this is an automated process, all files are exposed to the user for exploration.

### Input data

The input data for the scripts is largely based on work by Brinkerink *et al*.^8^ which consists of an openly available public global power system dataset as part of the work for the developed ‘PLEXOS-World’ power system model^34^. The dataset is composed from a wide range of public sources which, among others, includes global power plant capacities, power plant specific capacity factor time series for hydro, solar, and wind, historical electricity demand time series for all countries, and existing cross-border transmission capacities; refer to^8^ for a full overview of the dataset and all its used sources. Next to the above input data, the OSeMOSYS global scripts make use of tools for demand projections based on earlier work by Brinkerink *et al*.^35^ as well as transmission line specific cost projections for all global transmission pathways^36^. The OSeMOSYS Global scripts are directly connected to the PLEXOS-World dataset depositories and automatically extract the relevant data.

Additionally, OSeMOSYS Global pulls data from a variety of other sources. Capital costs and fixed operating costs for power plants are extracted from the World Energy Outlook^37^, while the variable operating costs are taken from the World Bank Commodity Market Outlooks^38^. Power plant operational lifespans are from the NREL Annual Technology Baseline^39^, while emission factors for each fuel are from the United States Environmental Protection Agency^40^. Finally, renewable resource potentials that determine the availability for future investments within the model are included for hydro^41^, solar^42^, and wind^43^.

### Model architecture

OSeMOSYS Global’s workflow can broadly be categorized into two processing steps. The first step is obtaining and formatting scenario data, while the second step is building, solving, and visualizing the scenario results. This section will describe these two steps in further detail.

#### Scenario creation

The core of OSeMOSYS Global is a series of Python scripts that reads in raw unformatted data and processes it into an OSeMOSYS compatible format based on user selected parameters. This is represented by the items in the first row of Fig. 7.

Firstly, the raw data is processed into formatted CSV files that hold data for the world (all 265 nodes). Included in these processing steps are creation of data related to electricity demand, timeslice structure, and generation technologies. Next, the user defined geographic filter is applied to create the scenario specific data.

**Fig. 8:**
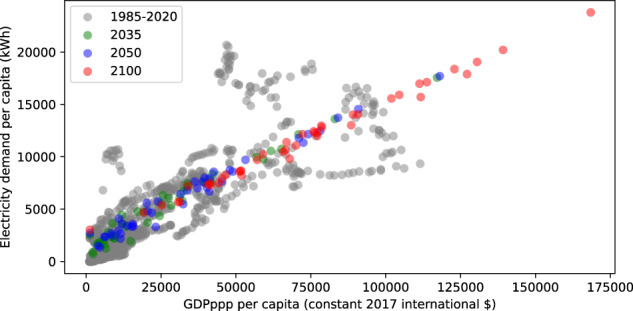
Demand Projections Sample Results for Asia. Grey Dots Represent Historical Country Level Values for Countries in Asia and the Coloured Dots Show Projected Values.

The first major component of the data processing involves demand forecasting. The “Demand Data” script is used to project electricity demand values which are based on a multivariate linear regression approach with GDP at purchasing power parity per capita and urbanization shares as independent variables, and electricity consumption per capita as the dependent variable. Details on this approach, including its limitations such as the fact that as of now the projection does not take into account factors that can affect demand projections, such as electrification, can be found at^35^. Moreover, the demand projection script creates a series of visualizations, with an example shown in Fig. 8. These graphs allow the modeller to confirm if the demand projections are reasonable and to identify outliers due to the demand projection algorithm. Note that by default countries are grouped at a continental level during the projection, however, users are free to group countries in a different manner for example by relative development status.

The next major data processing component involves creating power plants and their associated parameters. The “Power Plant Data” script creates a structured output of mining and powerplant technologies. A mining technology generates the raw resources that electricity generators use, such as natural gas, coal, wind, or water, to allow tracking of raw material usage. Power plant generators are the technologies that produce electricity, and will operate on one of the mined fuels. This script will also combine all plants of a given type in a single node together. This is needed because the input data specifies plants at an individual station level, while OSeMOSYS Global will represent all plants in a given region as one representative station to limit computational complexity. While the existing capacity of all technologies is included, resource limits on biomass, geothermal, and wave power were not implemented due to a lack of consistent global data on these resources. Instead, users are advised to consider the specific policy questions they wish to address and how specific resource constraints may impact these results.

Finally, the “Timeslice Data” script will generate the time slice structure defined by the user in the configuration file, and update parameters that rely on time slice definition respectively, such as the capacity factor and electricity demand profiles. OSeMOSYS Global uses representative days to timeslice the model^13,24^. This approach involves representing a time period using average values for variable parameters, such as loads or renewable generation profiles, over a specified time period. In OSeMOSYS Global, a representative period can be as fine as 24 hours per month (288 total time periods per year) or as coarse as one representative period per year. The model horizon can be set for any interval between the years of 2015 and 2100. With future model releases, the time slicing structure can be further enhanced through introducing more data or data clustering methods^26^.

#### Model processing

Once the scenario CSV data has been created, it is processed into an OSeMOSYS compatible datafile, the model is built and solved, and result data tables and graphics are generated. To transform the CSV data describing a specific scenario into an OSeMOSYS compatible datafile, the Python package OSeMOSYS Tools for Energy Work^44^ (otoole, https://github.com/OSeMOSYS/otoole), is used. The output datafile from otoole is fed into the open-source GLPK tool (https://www.gnu.org/software/glpk) with the OSeMOSYS model to create a solver independent linear programming file. This file can then be called by any solver described in the configuration file (CBC^45^, CPLEX^46^, or Gurobi^47^). Once solved, the solution file is processed by otoole to generate a complete set of result CSV files. The “Visualization” Python script will then process the CSV results to display a series of summary result tables and result graphs, some of which have been shown earlier in the Results section.

## Data Availability

All input datasets for the OSeMOSYS Global model are available from openly licensed sources as identified in the OSeMOSYS Global code repository (https://github.com/OSeMOSYS/osemosys_global). Data generated for the figures and analysis in this article can be replicated using version 0.4.0 of OSeMOSYS Global and modifying the configuration file accordingly.

## Code availability

All code for OSeMOSYS Global is provided in a public GitHub repository (https://github.com/OSeMOSYS/osemosys_global) under a MIT license. Instructions on how to install and run OSeMOSYS Global can be found in the repository or on the OSeMOSYS Global website (https://osemosys-global.readthedocs.io). While a basic understanding of Python and OSeMOSYS will be beneficial, it is not required to run the workflow. All users and readers are invited to contribute to OSeMOSYS Global following the contribution guidelines outlined on the repository.
